# Extreme root resorption in orthodontic practice: teeth do not have to be replaced with implants

**DOI:** 10.1590/2177-6709.24.5.020-028.oin

**Published:** 2019

**Authors:** Alberto Consolaro

**Affiliations:** 1 Universidade de São Paulo, Faculdade de Odontologia de Ribeirão Preto, Programa de Pós-Graduação em Odontopediatria (Ribeirão Preto/SP, Brazil).; 2 Universidade de São Paulo, Faculdade de Odontologia de Bauru (Bauru/SP, Brazil).

**Keywords:** Tooth resorption, Root resorption Apical resorption, Orthodontic movement.

## Abstract

The replacement of natural teeth that have extreme external apical root resorption, induced by orthodontic treatment, with osseointegrated implants is not justifiable biologically or clinically. These teeth should be preserved and keep their normal functions, as there is no greater mobility, pain or color change. They may undergo usual procedures, such as bleaching, restorations with veneers and other esthetic procedures that may be necessary along life. The pulp of these teeth is normal. If mobility of a tooth with extreme resorption is identified, the cause of mobility should be investigated, as it is not associated with resorption, not even at advanced stages. Tooth mobility may be associated with recent removal of orthodontic appliance, occlusal trauma, chronic inflammatory periodontal disease, or even severe cervical bone loss. In such cases, the cause of mobility should be eliminated and possible sequelae should be corrected, because these, and not root resorption, may actually require retention.

## RESUMO

A troca de dentes naturais com reabsorções inflamatórias externas apicais extremas, induzidas ortodonticamente, por implantes osseointegráveis não se justifica, biológica ou clinicamente. Esses dentes devem ser mantidos, cumprindo suas funções normais, pois não apresentam aumento de mobilidade, nem dor ou alteração de cor, podendo ser submetidos a procedimentos comuns, como clareamento, facetas e outras abordagens estéticas necessárias ao longo da vida. A polpa desses dentes é normal. Se os dentes com reabsorção extrema se apresentarem com mobilidade, deve-se buscar a causa dessa mobilidade, que não está associada à reabsorção, mesmo quando avançada. A mobilidade dentária deve estar relacionada com a recente remoção do aparelho ortodôntico, trauma oclusal, doença periodontal inflamatória crônica ou, ainda, com perda óssea cervical severa. Nesses casos, para eliminar a mobilidade, deve-se eliminar a causa e corrigir as sequelas, que - essas sim - podem necessitar de contenção, mas não pela reabsorção radicular.

Seven concepts are essential for the understanding of why osseointegrated implants should not be indicated to replace teeth with root resorptions due to orthodontic treatments:^1^ ^,^ ^2^

**Figure 1:**
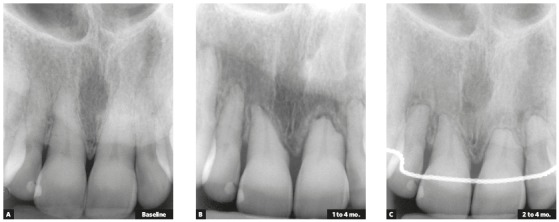
Extreme external inflammatory apical resorption under control; retention used, however, was unnecessary. In B, irregularity of active resorption, already corrected and interrupted in C (Source: Oliveira^2^, 2010).

1) The causes of tooth resorption are always local.
2) There are no systemic, endocrine or hereditary causes in the etiopathogeny of root resorptions. When a clinical cause is not found, the resorption is called idiopathic, which indicates that it was not possible to identify the cause despite all efforts made.
3) In human beings, there are no genes, diseases or ethnicity that makes people more susceptible to root resorptions. In other words, there is no individual, familial or racial susceptibility to the development of root resorptions during orthodontic treatment.
4) The only tooth resorption that is directly associated with orthodontic movements is external inflammatory apical resorption (Figs 1 to [Fig f2] [Fig f3] [Fig f4] [Fig f5] 6).
5) External cervical resorption and replacement resorption that occur during orthodontic treatment are the result of inadequate and traumatic procedures in the preparation of traction of unerupted teeth, often in the maxillary arch. The only cause of external cervical resorption and replacement resorption that occur during treatment without traction of unerupted teeth is traumatic tooth injury, particularly concussions.
6) Fifteen local predicting factors increase the probability of root resorption in orthodontic practice. Some are particularly important, such as variation in root and bone crest morphology, previous traumatic tooth injury, and several others that should be taken into consideration when planning each case.

**Figure 2 f2:**
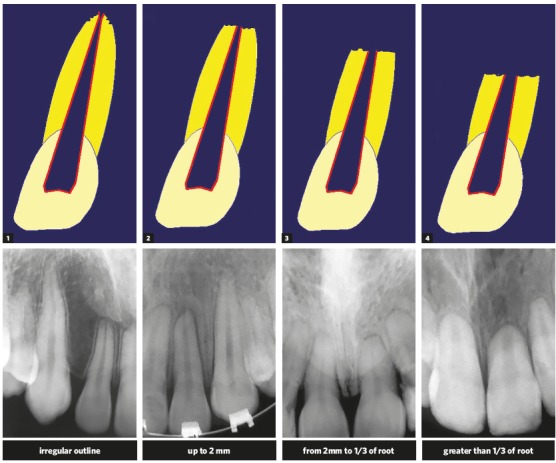
Classification of external inflammatory apical resorption in four degrees, devised for orthodontic practice (Source: Malmgren et al.^5^, 1982).

**Figure 3 f3:**
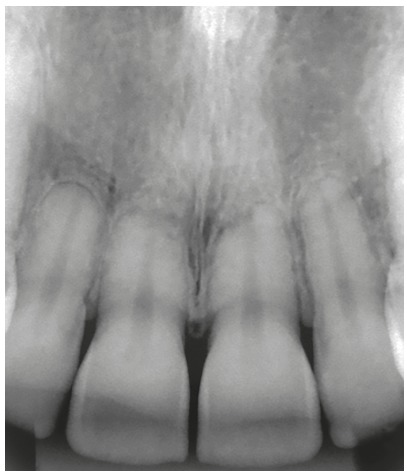
Active extreme external inflammatory apical resorption in the four maxillary incisors, associated with orthodontic treatment.

**Figure 4 f4:**
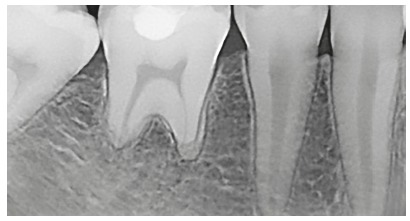
Active extreme external inflammatory apical resorption in first molar, associated with orthodontic treatment.

**Figure 5 f5:**
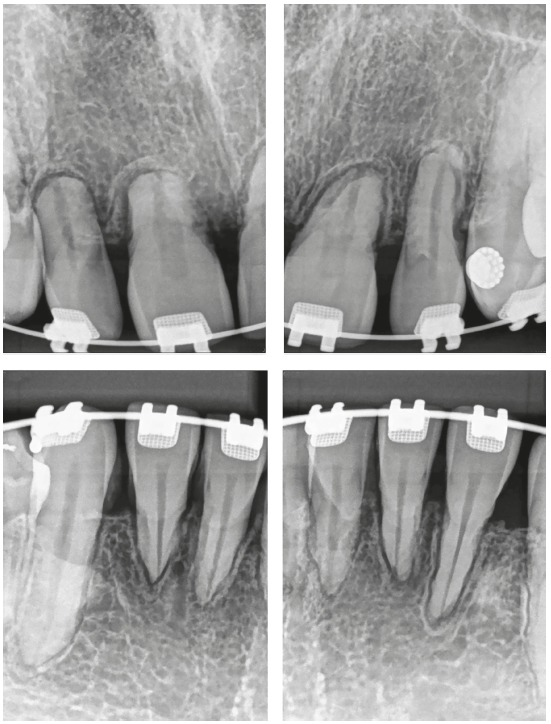
Active extreme external inflammatory apical resorption in maxillary and mandibular incisors, associated with orthodontic treatment.

**Figure 6:**
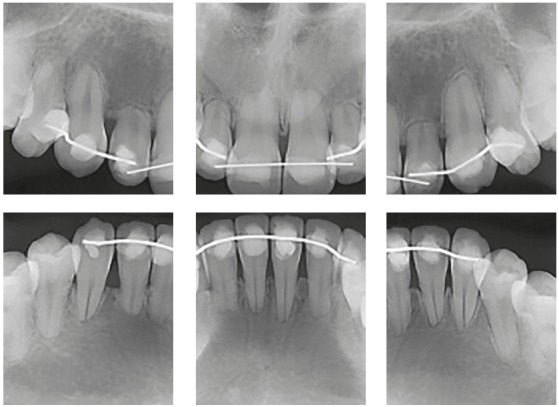
Active extreme external inflammatory apical resorption in four incisors, associated with orthodontic treatment. Retention, however, was unnecessary.

External inflammatory apical resorption is promoted by clasts and other cells found in the tissues of the periodontal ligament, without affecting any of the pulp tissues.

## MOST PERIODONTAL SUPPORT IS CERVICAL: THERE IS NO MOBILITY IN ROOT RESORPTION!

The root apex accounts for 10% of periodontal support. Losing the apical third does not lead to a significant loss of periodontal support. The middle third of the root is responsible for 30% of periodontal support, and a tooth with half a root still has more than 60% of its original periodontal support.

The cervical third of the root accounts for 60% of periodontal support because of its greatest diameter and circumference - probably to make up for the greater elasticity and deformation capacity of the cervical alveolar bone, which stabilizes the tooth and bone together.

In practice, a tooth with only one third of the cervical root may remain in the mouth and preserve its function in mastication, phonation and esthetics without any increased mobility or gingival changes.^3^

However, special care should be taken in the presence of this type of teeth. The patient should be convinced to:

1. Avoid holding food only with teeth, submitting them to excessive stress.
2. Wear an acrylic dental splint to sleep, thus avoiding bruxism or jaw tightening forces that act during sleep and only on a few teeth.
3. When practicing sports, wear a mouthguard, because even minor trauma may result in tooth avulsion.
4. If retention is applied, as an option or a cautionary measure, its purpose should be to increase the resistance of teeth with shortened roots in case of dental trauma in sports or in everyday life. Tooth mobility does not affect teeth with root resorption, not even in extreme cases.

If there is mobility in teeth with resorption, even in extreme cases, this will not be the result of resorption, but, rather, of:

» Still active or residual orthodontic forces applied.
» Active retention.
» Occlusal trauma affecting tooth with resorption.
» Iatrogenic orthodontic cervical bone loss.
» Severe cervical bone loss associated with chronic periodontal disease or occlusal trauma.

Such mobility should be corrected adequately and immediately after the precise determination of its cause.

## ORTHODONTIC TREATMENT SHOULD NOT BE CONDUCTED IN THIS CASE

**Figure 7:**
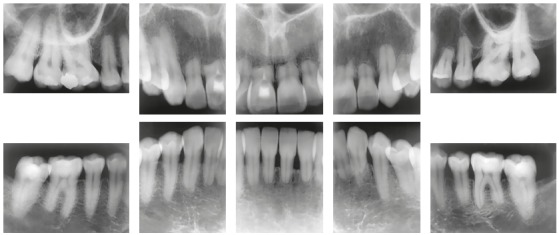
Extreme external inflammatory apical resorption associated with orthodontic treatment: endodontic treatment is not indicated, as observed in tooth #11. There are no systemic or hereditary causes for tooth resorptions in human beings.

**Figure 8:**
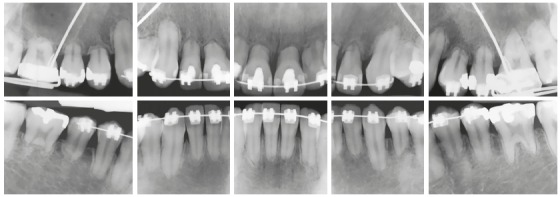
At four years of orthodontic treatment, no teeth with extreme inflammatory root resorption requires replacement with osseointegrated implant or endodontic treatment because they are vital, nor use of retention, because there is no mobility. Source: Consolaro and Furquim^3^, 2014.

**Figure 9:**
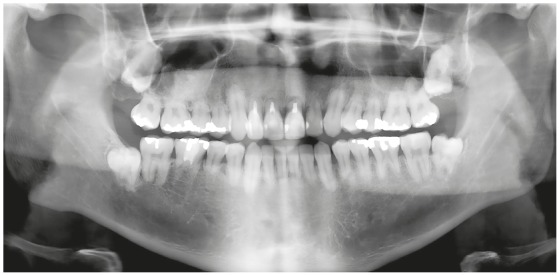
Endodontic treatment of three teeth that had extreme inflammatory root resorption after four years of orthodontic treatment was unnecessary, as shown in digital panoramic radiograph. Same clinical case as in Figure 8, at seven years of follow-up. Source: Consolaro and Furquim^3^, 2014.

The pulp of teeth with severely reabsorbed roots is vital^4^ and should never undergo endodontic treatment to control resorption or increase tooth resistance to resorption, because this will not happen (Figs 7, 8 and 9).

Numerous dentists, in cases of an emergency, lose control of their logical and biological reasoning and attempt to use all alternatives randomly, even those that are inappropriate and inefficient. 

Even when most of the roots have severe grade 4 resorption, according to Malmgren et al.^5^ (Fig. 2), no teeth should be extracted or undergo endodontic treatment. Such procedure does not make any difference in the prognosis of roots (Figs 7, 8 and 9). Tooth pulp is not involved in external resorption, and the medications injected in the canal do not affect the causes of resorption while it is maintained by active forces (Figs 1, 3 and 5). However, the removal of the forces applied interrupts resorption, and one week after removal no clasts are seen on the root surface anymore.

Resorption associated with orthodontic treatment may be controlled by removing the force applied. Seven days later, there will be no more clasts on the tooth root, and four to five weeks later, all the root surface will be regular and repaired with new cement and insertion of periodontal fibers. The periodontal ligament, and not the tooth pulp, is responsible for root resorption. It is useless to conduct orthodontic treatments under these restricted conditions. After six weeks, the length of the tooth with resorption will stabilize, with the formation of new cement, cementoblastic layer and periodontal ligament.

## IMPORTANT POINTS IN THE INTERPRETATION OF IMAGING STUDIES OF CERVICAL BONE IN EXTREME ROOT RESORPTION

Although it may sound incredible, periapical radiographs are more reliable than CT scans in these cases, when every small detail is important. 

Thin slices of cortical bone, even the most delicate bone trabeculae in thin areas, tend not to appear on CT scans and panoramic radiographs. Newer CT scanners, with better resolutions, may overcome this limitation. Several images may suggest that some teeth are held only by soft tissue, when, in fact, they are clinically stable and do not have any abnormal mobility. Three-dimensional CT scans are useful for the elucidation of the case only.

## MANAGEMENT PROTOCOL FOR CASES OF EXTREME EXTERNAL INFLAMMATORY APICAL RESORPTION ASSOCIATED WITH ORTHODONTIC TREATMENT

A tooth with external inflammatory apical resorption and only the cervical portion of the remaining root still has 60% of periodontal support. This tooth does not have to be replaced with an osseointegrated implant (Figs 8 and 9). To preserve teeth with extreme root resorption associated with orthodontic treatment, the parameters suggested by Consolaro and Furquim,^3^ in 2014, should be followed:

1) Teeth should be kept in the mouth for an indefinite time to preserve esthetics, without endodontic treatment, except when there are exclusively endodontic indications of treatment.
2) Occlusion should be carefully balance and have no interferences, which should be corrected as soon as detected.
3) Patient should wear a mouthguard to practice sports. In case of trauma to these shorter teeth, the protocols used should be the same as those adopted for teeth without shorter roots.
4) Biting hard and dense foods, such as whole fruits and bread, should be avoided.
5) In even mild or occasional cases of bruxism, the patient should routinely wear a dental splint to sleep, systematically.
6) Roots are very short, and tooth movements should be avoided.
7) A carefully devised treatment approach may be adopted if only orthopedic movements are required, without any orthodontic movements or anchorage that may affect these teeth, and if these planned changes do not lead to inflammation or stress of the periodontal ligament, which may trigger a new resorptive cycle.
8) Chronic inflammatory periodontal disease associated with bacterial plaque should be avoided by educating patients for optimal oral hygiene. Even minor cervical bone loss in these teeth is very harmful.
9) Teeth that are not fully or partially erupted should be removed, particularly when they are close to other teeth and may lead to root resorptions that may complicate the patient's condition due to orthodontic problems.
10) Parafunctional habits, such as fingernail biting, tongue thrust, object biting, and repetitive contacts with lip and tongue piercings, should be corrected and avoided.

## TOOTH IMPLANTS ARE NOT INDICATED TO REPLACE TEETH WITH EXTREME EXTERNAL APICAL RESORPTION INDUCED BY ORTHODONTIC TREATMENT: TEETH MAY KEEP THEIR NORMAL FUNCTIONS

If the steps listed in the topic above are taken, the chance of tooth loss is greatly reduced and reaches almost zero, and retention and replacement with osseointegrated implants are unnecessary. 

If mobility of a tooth with extreme resorption is identified, the cause of mobility should be investigated, as it is not associated with resorption, not even at advanced stages. Tooth mobility may be associated with recent removal of an orthodontic appliance, occlusal trauma, chronic inflammatory periodontal disease, or even severe cervical bone loss. In such cases, the cause of mobility should be eliminated and possible sequelae should be corrected, because these may actually require retention, although not because of root resorption. 

## EXTREME EXTERNAL INFLAMMATORY APICAL RESORPTION: IATROGENIC EVENT?

Most extreme external inflammatory apical resorptions are predictable in orthodontic practice, particularly when treatment planning includes the evaluation of 15 predicting factors.^12^

In some clinical cases, external inflammatory apical resorptions may be avoided if we consider these factors. In other mild or moderate cases that may also present with these predicting factors, resorptions may be inevitable if the plan is to achieve the previously defined clinical treatment objective, and are even “necessary” at times. 

Predictability allows us to establish a safe prognosis for each case and to find compatible orthodontic techniques for the patient’s biological conditions. To avoid cases of extreme or severe external inflammatory apical resorptions, the 15 predicting factors already known should be applied. 

Predictability is ensured by a safe prognosis and the control of root resorption during orthodontic treatment:

1. Analysis of periapical radiographs of all teeth or of a CT scan of the whole maxilla and mandible. Radiographs that show only the incisors do not provide information to extrapolate findings to the other teeth.
2. Predicting factors should be identified and included in planning.
3. Six to nine months after the appliance is placed, periapical radiographs or CT scans should be obtained again to evaluate root conditions.
4. At the end of the treatment, new periapical radiographs and CT scans should again be taken to evaluate root conditions before patient discharge.

Some cases are important exceptions:

1) Planning and treatment procedures, often adequate, do not count on patient collaboration in following all instructions: the way oral hygiene should be performed to preserve the appliance and the health of the mouth; punctuality and regularity of visits; patient life style and eating, sleeping and other general habits.
2) Extreme external inflammatory apical resorption associated with orthodontic treatment affects several teeth simultaneously. When resorption is found in only one or two teeth during orthodontic treatment, previous dental trauma should be suspected, as patients may forget to report the event when history is taken. Previous dental trauma is one of the main predicting factors of more severe resorptions during orthodontic movements.
3) The last exception, which is particularly important, refers to the way forces are applied during orthodontic treatment: there is still no technology or electronic devices to determine which force should be applied to each tooth! We still learn to apply the necessary force in the training received during our previous education in specialization courses in Orthodontics. There is no other method to train a skillful orthodontist, and even experienced, well-trained orthodontists have their cases of severe external inflammatory apical resorption.

The most efficient way to avoid extreme external inflammatory apical resorption is to obtain periapical radiographs or CT scans of the teeth at the times recommended: in cases in which extreme external inflammatory apical resorption is likely to occur, they may be detected if images are obtained at six and nine months after the beginning of orthodontic treatment. Once forces are controlled, resorptions stop, periodontal tissues regenerate and return to their normal state five to six weeks later.

## FINAL CONSIDERATIONS

The replacement of natural teeth with extreme external apical root resorption with osseointegrated implants is not justifiable biologically or clinically. These teeth should be preserved and keep their normal functions, as there is no greater mobility, pain or color change. They may undergo usual procedures, such as bleaching, restorations with veneers and other esthetic procedures that may be necessary along life. The pulp of these teeth is normal.
